# Monovalent and Divalent Designs of Copper Radiotheranostics Targeting Fibroblast Activation Protein in Cancer

**DOI:** 10.3390/cancers16244180

**Published:** 2024-12-15

**Authors:** Pawan Thapa, Sashi Debnath, Anjan Bedi, Madhuri Parashar, Paulina Gonzalez, Joshua Reus, Hans Hammers, Xiankai Sun

**Affiliations:** 1 Department of Radiology, University of Texas Southwestern Medical Center, Dallas, TX 75390, USA; ppawann86@gmail.com (P.T.); sashi.debnath@utsouthwestern.edu (S.D.); anjan.bedi@utsouthwestern.edu (A.B.); paulina.gonzalez@utsouthwestern.edu (P.G.); 2 Department of Internal Medicine, University of Texas Southwestern Medical Center, Dallas, TX 75390, USA; madhurriparashar@gmail.com (M.P.); joshua.reus@utsouthwestern.edu (J.R.); hans.hammers@utsouthwestern.edu (H.H.); 3 Kidney Cancer Program, University of Texas Southwestern Medical Center, Dallas, TX 75390, USA; 4 Advanced Imaging Research Center, University of Texas Southwestern Medical Center, Dallas, TX 75390, USA

**Keywords:** fibroblast activation protein, copper radiopharmaceuticals, cross-bridged cyclam, theranostic, positron emission tomography, renal cell carcinoma

## Abstract

**Background**: Fibroblast activation protein (FAP)-targeted theranostic radiopharmaceuticals have shown desired tumor-to-background organ selectivity due to the ubiquitous presence of FAP within the tumor microenvironment. However, suboptimal tumor retention and fast clearance have hindered their use to deliver effective cancer therapies. With well-documented FAP-targeting moieties and linkers appending them to optimal chelators, the development of copper radiopharmaceuticals has attracted considerable interest, given the fact that an ideal theranostic pair of copper radionuclides (^64^Cu: t_1/2_ = 12.7 h; 17.4% β^+^; E_β_^+^_max_ = 653 keV and ^67^Cu: t_1/2_ = 2.58 d; 100% β^−^; E_β_^−^_max_ = 562 keV) are available. Herein, we report our design, synthesis, and comparative evaluation of monovalent and divalent FAP-targeted theranostic conjugates constructed from our previously reported bifunctional chelator scaffold (BFS) based on 1,4,8,11-tetraaza-bicyclo [6.6.2]hexadecane-4,11-diacetic acid (CB-TE2A), which forms the most stable complex with Cu(II). **Methods**: After synthesis and characterization, the monovalent and divalent conjugates were radiolabeled with ^64^Cu for in vitro cell assays, followed by in vivo positron emission tomography (PET) imaging evaluation in relevant mouse models. **Results**: Both ^64^Cu-labeled conjugates showed high in vitro stability and anticipated FAP-mediated cell binding and internalization. The divalent one showed significantly higher FAP-specific tumor uptake than its monovalent counterpart. **Conclusions**: Our results demonstrate that the BFS-based multivalent approach can be practically used to generate FAP-targeted radiotheranostic agents for effective cancer diagnosis and treatment.

## Simple Summary

The growing interest in the development of fibroblast activation protein (FAP)-targeted radiotheranostics is sustained by their potential applicability for precision therapy of epithelial malignancies. However, tumor uptake retention and effective clearance from normal tissues remain to be addressed for further translational planning and clinical use. In this work, we report an effective strategy to present an FAP-targeting moiety on a bifunctional chelator scaffold (BFS) reported by us to optimize the design of FAP-targeted copper radiopharmaceuticals. Biological studies using clinically relevant in vitro and in vivo models demonstrate that the multi-presentation approach enabled by the BFS has great potential to achieve the desired therapeutic index for FAP-targeted radiotheranostics.

## 1. Introduction

The tumor microenvironment (TME) plays a crucial role in shaping the complex and diverse tumor ecosystem [1]. Despite this understanding, there have been many obstacles in selectively targeting cancer-associated fibroblasts (CAFs), the main regulator in the construction and reconstruction of the TME [2,3]. A crucial component of desmoplastic tumors, CAFs have long been a research focus in oncology. It has been documented that CAFs produce extracellular matrix, nutrients, cytokines, chemokines, and other signaling molecules, which support their roles in the TME. Activated CAFs express fibroblast activation protein (FAP), a type II transmembrane cell surface proteinase [4], which is profoundly activated during tissue remodeling, tumor growth, and inflammatory disorders. More than 90% of epithelial malignancies, including breast, pancreatic, lung, prostate, and colorectal cancer, are observed with upregulated expression of FAP [5]. The broad spectrum of FAP expressions in various malignancies has prompted numerous investigations on the pro- and anti-tumor impacts of FAP activities. It is known that FAP expression regulates tumor growth through a variety of mechanisms, including tumor cell proliferation, epithelial-to-mesenchymal transition, angiogenesis, drug resistance, and immunosuppression [6]. Therefore, FAP presents a unique opportunity to intervene in solid tumors and stromal constituents with targeted theranostics [7,8,9,10].

**Figure 1:**
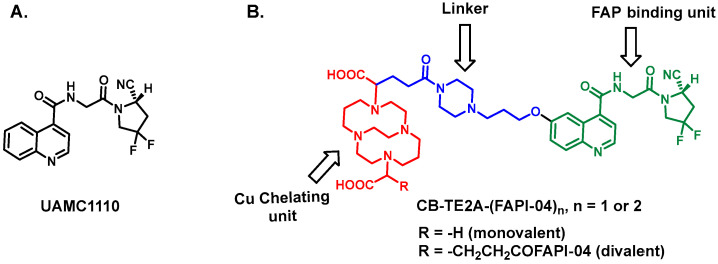
(**A**) UAMC1110, lead structure for FAPI development; (**B**) multivalent conjugate design for FAP-targeted copper radiotheranostics.

Early efforts of targeting FAP for cancer treatment started with radioimmunotherapy using radiolabeled antibodies (e.g., ^131^I-mAbF19). However, the extended circulation time of intact antibodies impeded the delivery of effective radiation doses to the target [11]. Therefore, considerable efforts had shifted to developing small organic molecules for FAP inhibition. As a result, quinoline-derived small-molecule inhibitors emerged as the most effective FAP inhibitors (FAPIs), and their uses have been advanced to FAP-targeted cancer imaging with positron emission tomography (PET) and single-photon emission computer tomography (SPECT) [12,13]. One such example is UAMC1110 (Figure 1A), a gold standard inhibitor of choice for the development of FAP-targeted radiotheranostics [5]. To date, chemical modification of the quinoline group in UAMC1110 has afforded a variety of FAPI conjugates with metal chelators, such as DOTA (1,4,7,10-tetraazacyclododecane-1,4,7,10-tetraacetic acid) and NOTA (1,4,7-triazacyclononane-N, N′,N″-triacetic acid), which can be labeled with diagnostic and/or therapeutic radionuclides for the desired purposes. Currently, the most potent theranostic conjugates are developed from FAPI-02, FAPI-04, FAPI-46, FAPI-74, and OncoFAP [14,15]. For instance, in a comparative study [^68^Ga]Ga-DOTA-FAPI-04 outperformed 2-deoxy-2-[^18^F]fluoroglucose ([^18^F]FDG) in PET imaging of primary gastric cancer and its metastatic lesions [15]. Due to the tremendous success of the radiotheranostic pair of [^68^Ga]Ga-PSMA-11 and [^177^Lu]Lu-PSMA-177, which target prostate-specific membrane antigen (PSMA), in the clinical management of patients with metastatic castration-resistant prostate cancer [16], such ^68^Ga and ^177^Lu paired radiotheranostics have set the benchmark for the development of FAP-targeted radiotheranostics in preclinical and clinical studies [14]. For example, a pair of ^68^Ga- and ^177^Lu -labeled FAPI-04 has been examined for their diagnostic and therapeutic applications in preclinical and clinical studies [17,18]. However, the radionuclides used in these theranostic pairs [18,19] are different metal ions with strikingly different half-lives (e.g., ^68^Ga: t_1/2_ = 68 min vs. ^177^Lu: t_1/2_ = 6.7 d). In other words, they are not ideally suited for paired radiotheranostic applications. As such, additional radionuclide pairs have been explored [20,21,22,23]. Theranostic conjugates with chemically identical radionuclides such as yttrium (^86^Y/^90^Y), iodine (^123/124^I/^131^I), and copper (^64^Cu/^67^Cu) offer advantages including identical pharmacokinetics and biodistribution, thus enabling precision dosimetry for personalized treatment strategies in addition to the same chemical synthesis facilitating translational study planning [24,25]. However, the observed high non-target organ uptake and low tumor retention limited their further application mainly due to the suboptimal in vivo stability of the metal chelate moiety and rapid clearance of the FAPI conjugates [21].

Therefore, considerable attention has been drawn to optimize the chelator/ligand design for stable metal radiopharmaceuticals [26]. Our previous work on the cross-bridged cyclam derivative 1,4,8,11-tetraaza-bicyclo[6.6.2]hexadecane-4,11-diacetic acid (CB-TE2A) [27,28] has demonstrated the optimal chelation of CB-TE2A to ^64^Cu^2+^ with dramatically enhanced in vivo stability and effective clearance from non-target organs as compared to the commonly used DOTA and NOTA, as well as their analogs [29]. Alternatively, sarcophagine-based chelators like DiAmSar offer comparable binding ability to Cu(II) ions and in vivo stability of the overall complex [30]. However, direct conjugation of biomolecules with primary amine in the presence of more reactive secondary amines of DiAmSar is not straightforward [31]. In addition to forming an inert complex with Cu(II), CB-TE2A offers a suitable platform after a chemical modification to become a bifunctional chelator scaffold (BFS) [32]. The BFS is capable of presenting multiple copies of targeting molecules to maximize the desired tumor retention of copper radiopharmaceuticals [27]. In this context, we have seen that the use of “polyvalent effect” and/or “albumin hitchhiking” has become an effective strategy to improve the desired diagnostic and therapeutic efficacies of radiotheranostic agents [33,34,35].

In this work, we present the use of a multivalent BFS derived from CB-TE2A to display one and two copies of FAPI-04, a well-validated FAPI, for the construction of monovalent and divalent FAP-targeted radiotheranostics (Figure 1B). Comparative studies were performed in both in vitro and in vivo models when labeled with ^64^Cu to assess the desired features of the radiotheranostic agents.

## 2. Materials and Methods

### 2.1. General Materials and Procedures

Chemicals were acquired from Sigma-Aldrich, Inc. (St. Louis, MO, USA), BroadPharm (San Diego, CA, USA), and Fisher Scientific (Hampton, NH, USA) and used directly without further purification. Reactions were performed under atmospheric conditions unless otherwise mentioned. Silica gel-coated aluminum plates (EMD Merck F254) were used for thin-layer chromatography (TLC) and observed under a 254 nm UV lamp or with chemical stains (Ninhydrin, Iodine etc.) for relevant reactions.

Agilent Prep-C18 column (150 × 21.2 mm, 5 μm) (Santa Clara, CA, USA) installed in an Agilent 1260 Infinity Prep HPLC system inbuilt with 1260 photodiode array detector was utilized for reverse-phase semi-preparative high-performance liquid chromatography (HPLC). Liquid chromatography–mass spectrometry (LC-MS) were performed on an Agilent 6540 Accurate-Mass Quadrupole Time-of-Flight LC-MS system endowed with a 1290 ultra-performance liquid chromatography (UPLC) system (Santa Clara, CA, USA). Radiolabeled compounds were characterized on C18 Atlantis^®^ T3 column (4.6 × 250 mm, 5 µm) (Waters, Ireland) installed in an Agilent 1220 Infinity II analytical HPLC system connected to a 1220 LC diode array detector (DAD) (76337 Waldbronn, Germany) and an in-line Eckert and Ziegler radio detector (Eckert and Ziegler Radiopharma, Inc., Hopkinton, MA, USA). In vitro assays were quantified with the radioactivity counts obtained from a 2480 PerkinElmer automatic gamma counter (Waltham, MA, USA). Milli-Q water, obtained from a Millipore Gradient Milli-Q water system (Burlington, MA, USA) was used to prepare aqueous buffer solutions. Our previously reported method was adapted for the synthesis of CB-TE2A(*^t^*Bu)_2_-COOH and CB-TE2A(*^t^*Bu)_2_-(COOH)_2_ [27]. FAPI-04 was produced following a reported method with minor modifications [12,13].

GraphPad Prism 10.4.0 (GraphPad Inc., San Diego, CA, USA) was utilized to conduct statistical investigations and assemble graphs. A *p*-value of < 0.05 (unpaired *t*-test) was deemed statistically significant. ChemDraw 23.1.1 (Waltham, MA, USA) was utilized to demonstrate all the chemical structures and synthetic routes.

Abbreviations: N,N,N′,N′-tetramethyl-O-(1H-benzotriazol-1-yl)uronium hexafluorophosphate (HBTU), N,N-diisopropylethylamine (DIPEA), dimethylformamide (DMF), trifluoroacetic acid (TFA), phosphate-buffered saline (PBS), Dulbecco’s modified eagle medium (DMEM), ethylenediaminetetraacetic acid (EDTA), 4′,6-diamidino-2-phenylindole (DAPI), diethylenetriaminepentaacetic acid (DTPA), trifluoroacetic anhydride (TFAA), p-toluenesulfonic acid (p-TSA), dichloromethane (DCM), dimethyl sulfoxide (DMSO), patient-derived xenografts (PDX), acetonitrile (ACN), counts per minute (CPM), mass spectrometry using electrospray ionization (MS (ESI)), molar (M), millimolar (mM), ammonium acetate (NH_4_OAc), carbon dioxide (CO_2_), hydrochloric acid (HCl), percent injected dose per gram (% ID/g), polyethylene glycol sorbitan monolaurate (TWEEN^®^ 20), phosphate-buffered saline with 0.05% TWEEN^®^ 20 (PBST), post-injection (p.i.), standard deviation (s.d.), computed tomography (CT).

### 2.2. Chemistry

#### 2.2.1. Synthesis of [(S)-N-(2-(2-cyano-4,4-difluoropyrrolidin-1-yl)-2-oxoethyl)-6-(3-(4-tert-butoxycarbonylpiperazin-1-yl)propoxy)quinoline-4-carboxamide] (Boc-FAPI-04)

To a 5 mL Schlenk flask, 6-(3-(4-*tert*-butoxycarbonylpiperazin-1-yl)-1-propoxy)quinoline-4-carboxylic acid (16.7 mg, 0.040 mmol), HBTU (17.1 mg, 0.045 mmol) and (*S*)-4,4-difluoro-1-glycylpyrrolidine-2-carbonitrile (10.8 mg, 0.057 mmol) were added in 500 μL of DMF followed by DIPEA (27.5 μL, 0.160 mmol). The resulting mixture was stirred for 16 h under nitrogen atmosphere at room temperature and monitored by LC-MS. Then, the mixture was quenched with 0.5 mL water and purified by reverse-phase HPLC using a binary gradient method within the range of ACN/H_2_O (1/4) to ACN/H_2_O (7/3) in 25 min; the HPLC eluents were modified with 0.1% TFA. Lyophilization of the relevant pure fraction afforded 15.0 mg (0.025 mmol; 64%) of Boc-FAPI-04 as a pale-yellow solid. MS (ESI) *m/z* calcd: 586.27; found: 587.13 ([M + H]^+^); Figures S1 and S2.

#### 2.2.2. Synthesis of CB-TE2A(*^t^*Bu)_2_-FAPI-04

Deprotection of Boc-FAPI-04 was conducted by treating Boc-FAPI-04 (7.0 mg, 0.012 mmol) with excess *para*-toluenesulfonic acid monohydrate (19.2 mg, 0.10 mmol) in acetonitrile at 40 °C for 3 h. The product formation was confirmed by LC-MS analysis of the crude reaction mixture, and the residual solvent was removed under reduced pressure in a rotary evaporator. The resulting crude FAPI-04 [36] was directly used for the subsequent conjugation reaction by adding it to the mixture of CB-TE2A(*^t^*Bu)_2_-COOH (6.0 mg, 0.009 mmol), HBTU (3.75 mg, 0.01 mmol), and DIPEA (9.0 µL, 0.05 mmol) in 500 µL of DMF. The resulting mixture was then stirred at room temperature for 24 h under nitrogen atmosphere. DMF was removed under reduced pressure, and the crude product was reconstituted in acetonitrile followed by reverse-phase HPLC purification starting with ACN/H_2_O (1/4) and ending with ACN/H_2_O (4/1) in 25 min; all HPLC eluents contained 0.1% TFA. Lyophilization of the collected pure fraction gave 4.0 mg (0.004 mmol; 40%) of the desired product CB-TE2A(*^t^*Bu)_2_-FAPI-04 as white solid. MS (ESI) *m/z* calcd for C_51_H_76_F_2_N_10_O_8_: 994.58; found 995.45 [M + H]^+^.

#### 2.2.3. Synthesis of CB-TE2A-FAPI-04

Monovalent CB-TE2A(*^t^*Bu)_2_-FAPI-04 (3.3 mg, 0.003 mmol) was reacted with TFA (200 µL) at room temperature for 3 h. After evaporation of the reaction mixture, the crude residue was purified by reverse-phase HPLC (10% acetonitrile/90% H_2_O to 60% acetonitrile/40% H_2_O in 30 min; all solvents contained 0.1% TFA). Lyophilization of the collected fraction gave 2.7 mg (0.003 mmol; 94%) of the desired conjugate CB-TE2A-FAPI-04 as white solid. MS (ESI) *m/z* calcd for C_43_H_60_F_2_N_10_O_8_: 882.46; found 883.49 [M + H]^+^; Figures S3 and S4.

#### 2.2.4. Synthesis of CB-TE2A(*^t^*Bu)_2_-(FAPI-04)_2_

In a two-neck Schlenk flask, CB-TE2A(*^t^*Bu)_2_-(COOH)_2_ (4.8 mg, 0.008 mmol), HBTU (6 mg, 0.015 mmol), and DIPEA (14.6 µL, 0.08 mmol) in 500 µL DMF were stirred at room temperature for 5 min under nitrogen atmosphere. Crude FAPI-04 (14 mg, 0.028 mmol) was added to this reaction mixture. The reaction mixture was stirred at room temperature for 24 h. The volatiles were removed under vacuo, and the crude product was reconstituted in acetonitrile followed by the purification via reverse-phase HPLC using a binary gradient method within the range of ACN/H_2_O (1/4) to ACN/H_2_O (4/1) in 28 min; all solvents were modified with 0.1% TFA. Lyophilization of the relevant pure fraction furnished 3.0 mg (0.002 mmol; 25%) of the desired product CB-TE2A(*^t^*Bu)_2_-(FAPI-04)_2_ as pale-yellow solid. MS (ESI) *m/z* calcd for C_72_H_106_F_4_N_16_O_12_: 1534.81; found 1535.79 [M + H]^+^.

#### 2.2.5. Synthesis of CB-TE2A-(FAPI-04)_2_

Divalent CB-TE2A(*^t^*Bu)_2_-(FAPI-04)_2_ (2.2 mg, 0.0015 mmol) was charged with TFA (200 µL) at room temperature, and the mixture was stirred for 3 h. After removing the volatiles in vacuo, the crude residue was purified by reverse-phase HPLC using a binary gradient method within the range of ACN/H_2_O (1/9) to ACN/H_2_O (7/3) in 25 min; all solvents were modified with 0.1% TFA. Lyophilization of the relevant pure fraction gave 1.6 mg (0.001 mmol; 75%) of the desired conjugate CB-TE2A-(FAPI-04)_2_ as white solid. MS (ESI) *m/z* calcd for C_70_H_90_F_4_N_16_O_12_: 1422.69; found 1423.51 [M + H]^+^; Figures S5 and S6.

### 2.3. Radiochemistry

Preparation of [^64^Cu]Cu-CB-TE2A-FAPI-04 was carried out by mixing and incubating 2 μg of monovalent precursor conjugate in 200 μL of 0.4 M NH_4_OAc (pH = 6.5) buffer with 130 MBq of [^64^Cu]CuCl_2_ at 75 °C for 0.5 h. To this reaction vial, 5 μL of 5 mM DTPA solution was added. The resulting mixture was left at room temperature for 5 min to remove non-specifically bound ^64^Cu. The purification of [^64^Cu]Cu-CB-TE2A-FAPI-04 was performed by passing the mixture through a Sep-Pak C-18 plus cartridge. After rinsing the cartridge two times with 5 mL water, [^64^Cu]Cu-CB-TE2A-FAPI-04 was eluted with 1 mL ethanol. The product was analyzed by radio-HPLC to determine the radiochemical purity. For ^64^Cu-labeling of CB-TE2A-(FAPI-04)_2_, 5 μg of the divalent conjugate in 10 μL DMSO was added to 150 μL of 0.4 M NH_4_OAc (pH = 6.5) buffer containing 296 MBq of ^64^CuCl_2_. The reaction mixture was incubated at 75 °C for 0.5 h, followed by adding 5 μL of 5 mM DTPA solution and a 5 min incubation at room temperature. The purification and characterization of [^64^Cu]Cu-CB-TE2A-(FAPI-04)_2_ were performed using the same procedures as described above for [^64^Cu]Cu-CB-TE2A-FAPI-04.

### 2.4. Serum Stability Study

In vitro stability of [^64^Cu]Cu-CB-TE2A-FAPI-04 and [^64^Cu]Cu-CB-TE2A-(FAPI-04)_2_ was analyzed after incubating with human serum. For this purpose, 100 µL of human serum was added with an aliquot (~1.85 MBq in 20 µL PBS) of [^64^Cu]Cu-CB-TE2A-FAPI-04 or [^64^Cu]Cu-CB-TE2A-(FAPI-04)_2_. The mixtures were then incubated at 37 °C for 1, 4, and 24 h. Following precipitation of the serum proteins with 420 µL of ethanol, the vials were centrifuged for 5 min at a speed of 14,000 rpm. The supernatant was purified with a 0.2 µm filter, transchelated ^64^Cu would stay with the protein trapped in the filter, and the radiochemical purity of [^64^Cu]Cu-CB-TE2A-FAPI-04 or [^64^Cu]Cu-CB-TE2A-(FAPI-04)_2_ was further analyzed by radio-HPLC of the supernatant.

### 2.5. Lentivirus Production

The HEK293T cells (2–3 × 10^6^) were seeded per 100 cm^2^ plate with 9 mL DMEM and incubated for 24 h in a humidified incubator at 37 °C with 5% CO_2_. The following day, 20 μL pPackH1, 75 μL Fu Gene, 3.5 μg FAP plasmid, and 800 μL serum-free DMEM were mixed in a conical tube using a vortex mixer for 10 sec followed by incubation at room temperature for 15 min. The mixture was poured onto the cells, and the plate was set in the incubator for 72 h. The cell medium was collected and centrifuged for 15 min at 3000× *g* to pellet cell debris. The supernatant was transferred to a fresh tube, and PEG-It was added to reach a final concentration of 1×. The resultant mixture was then incubated at 4 °C for 24–72 h. The mixture was centrifuged at 4 °C, 1500× *g* for 15 min and used for transduction.

### 2.6. Transduction

The RENCA cells (5 × 10^5^) were seeded in a 24-well plate with 1 mL DMEM media and incubated overnight at 37 °C with 5% CO_2_. A total of 100–500 μL virus and 1.5 μL 1000× polybrene were introduced. Cells were evaluated daily and expanded as needed. Immunofluorescence labeling was used to confirm the presence of FAP^+^ RENCA cells.

### 2.7. Immunofluorescence Staining

In a 96-well plate, 10,000 cells (RENCA-FAP, and RENCA) were seeded in each well with 200 μL DMEM and incubated overnight at 37 °C with 5% CO_2_. Following the removal of the media, the cells were fixed in 150 μL of 10% formalin for 15 min at room temperature. Once the formalin was removed, the cells were rinsed with 150 μL of 1× PBS before being blocked for 1 h at room temperature with 1% BSA. After removing the BSA, 150 μL fluorescent antibody (R&D-FAB3715G) diluted to 1:1000 in 1% BSA was added and incubated overnight at 4 °C. After that, the antibody was removed and washed in 1× PBS. The 150 μL DAPI was diluted to 1:50 in 1× PBS. Cells were examined under a fluorescent microscope after about 10 min of incubation in the dark.

### 2.8. RENCA-FAP Immunofluorescence Staining

Slides were warmed for 10 min in a 60 °C oven before deparaffinization. The deparaffinization and rehydration process involved incubating formalin-fixed paraffin-embedded tumor tissue in a series of solvents—xylene (3 × 5 min) followed by ethanol gradients of 100% ethanol (2 × 2 min), 95% ethanol (2 × 2 min), 70% ethanol (2 × 2 min), and 50% ethanol (2 × 2 min), ending with 1x PBS (1 × 3 min). For antigen retrieval, a buffer containing 10 mM Tris-HCl, 1 mM EDTA, and 10% glycerol (pH 9) was prepared. Slides were immersed in the buffer and heated at 115 °C for 15 min in a pressure cooker (Biocare Medical) filled with 500 mL of water. Then, the slides were cooled at room temperature for 30 min and rinsed with 1× PBS. Sections of tissues were enclosed by a hydrophobic barrier created with a PAP pen and blocked for 10 min with 2.5% horse serum (Vector Laboratories, CA, USA). Incubation with FAP antibody (R&D-FAB3715G) was performed on a shaker at 4 °C overnight. The washing procedure consisted of one rinse with PBST (0.2% Tween 20 and 2 mM EDTA) and two additional rinses with PBST (0.05% Tween 20 and 2 mM EDTA), each for 5 min. For nuclear counterstaining, 4′,6-diamidino-2-phenylindole (DAPI) was incorporated. Slides were cover slipped using ProLong Gold (no. P36931; Life Technologies). Slides were scanned with a Leica Microscope (Leica Microsystems Inc., Deerfield, IL, USA). Images were acquired with DAPI and AF488 channels at 20× and 40× magnifications. During image analysis in Fiji software, all images were thresholded uniformly across stains to maintain consistency.

### 2.9. Cell Culture and Animal Model

Animal experiments were carried out according to the existing animal protocol (APN: 2020-102851; effective from 24 May 2024 to 1 February 2026) authorized by the Institutional Animal Care and Use Committee (IACUC) at the University of Texas Southwestern Medical Center. The RENCA cell line was transduced with the lentivirus-containing fibroblast activation protein (FAP) gene. The RENCA-FAP and RENCA cells were cultured in DMEM high-glucose media (D5671, sigma-aldrich) with 10% fetal bovine serum, penicillin/streptomycin, and 1 µg/mL of puromycin. All the cells were expanded at 37 °C under 5% CO_2_ for tumor development and passaged at 75–90% confluency. Cells were injected subcutaneously (1.0 × 10^6^ cells in 100 µL Hank’s buffered salt solution (HBSS)) into the shoulders of male BALB/C mice (6–8 weeks). XP-185 (patient-derived xenografts, PDX) tumor tissues were implanted into the right shoulders of severe combined immunodeficiency (SCID) mice (6–8 weeks). The mice were housed in laminar flow cages at ~22 °C with ~55% relative humidity in a light/dark cycle of 12 h. Mice had free access to autoclaved water and commercial food, and they were checked every other day for general observations and tumor burdens.

### 2.10. Cell Uptake Assay

The cell-binding potency of [^64^Cu]Cu-CB-TE2A-FAPI-04 and [^64^Cu]Cu-CB-TE2A-(FAPI-04)_2_ was evaluated with RENCA-FAP (FAP^+^) and RENCA (FAP^−^) cells. Cells were seeded in 6-well plates of an average number of ∼1 × 10^6^ cells per well (n = 3) and set in a humidified incubator overnight at 37 °C with 5% CO_2_. Afterward, a binding buffer (20 mM Tris, 150 mM NaCl, pH 7.4) was added to rinse the cells twice. The cells were then incubated for 1 h at 37 °C with [^64^Cu]Cu-CB-TE2A-FAPI-04 or [^64^Cu]Cu-CB-TE2A-(FAPI-04)_2_ (∼5 × 10^5^ CPM) in a 500 μL binding buffer. For the analysis of non-specific binding, RENCA-FAP (FAP^+^) and RENCA (FAP^−^) cells in 6-well plates were incubated for 1 h with FAP-targeting ligand FAPI-04 (1 mM), followed by the addition of [^64^Cu]Cu-CB-TE2A-FAPI-04 or [^64^Cu]C-CB-TE2A-(FAPI-04)_2_ (∼1 × 10^6^ CPM). A cold binding buffer was added to rinse the cells three times. Cells were then stimulated at 37 °C with 0.5 mL of 1 M NaOH for 15 min. The NaOH-digested cells were collected in glass tubes for gamma counting.

### 2.11. Internalization Assay

An internalization assay was performed with RENCA-FAP (FAP^+^) cells. Briefly, 18-well plates were seeded with 2.0 × 10^5^ RENCA-FAP cells per well and set in a humidified incubator overnight at 37 °C with 5% CO_2_. Afterward, a 500 µL binding buffer (20 mM Tris, 150 mM NaCl, pH 7.4) was added to wash the cells. Then, [^64^Cu]Cu-CB-TE2A-FAPI-04 or [^64^Cu]Cu-CB-TE2A-(FAPI-04)_2_ (∼4 × 10^5^ CPM) in a 500 μL binding buffer was added to each well and incubated for 3, 10, 30, 60, 120, and 180 min (n = 3). After each time point, the cells were washed with ice-cold binding buffer and incubated for 5 min with 0.5 mL ice-cold stripping buffer (150 mM NaCl, 50 mM glycine, pH 3.0) to remove surface-bound radiolabeled conjugate. Cells were then stimulated at 37 °C with 0.5 mL of 1 M NaOH for 15 min to solubilize, to collect internalized [^64^Cu]Cu-CB-TE2A-FAPI-04 or [^64^Cu]Cu-CB-TE2A-(FAPI-04)_2_. The radioactivity of both surface-bound and internalized radiotracers was counted.

### 2.12. Western Blotting and IHC Method

Western blotting and immunohistochemical staining were performed on XP-185 tumor xenograft using FAP-α antibody (ab53066, Abcam, Waltham, MA, USA). The details of these procedures were adopted from our previously reported procedure [37].

### 2.13. Small Animal PET/CT Imaging

The PET/CT scans were performed in mice when tumors became palpable using a Mediso NanoScan PET/CT system (Mediso Medical Imaging Systems, Budapest, Hungary) equipped with a 4-mouse bed and a heating chamber. Mice bearing XP-185 (n = 4) and FAP^+^ RENCA-FAP (n = 4) tumors were anesthetized by 2% isoflurane with O_2_ before the scan, and the anesthesia was maintained during the dynamic (0–60 min, for XP-185 tumor-bearing mice) and/or static PET out to 24 h p.i. data acquisition followed by intravenous injection of 4–5 MBq of [^64^Cu]Cu-CB-TE2A-FAPI-04 or [^64^Cu]Cu-CB-TE2A-(FAPI-04)_2_ in <150 µL of PBS [38]. After PET data acquisition, an 8 min CT scan was performed at each time point to obtain anatomical information. The PET acquisition data were reconstructed using the Tera-Tomo™ 3D PET iterative algorithm and real-time Monte Carlo-based physical modeling. Statistical analysis was performed after the PET, and CT images were co-registered for various regions of interest (ROIs). Radiotracer accumulation in ROIs was measured as a percentage of injected dose per gram of tissue (%ID/g). Unlabeled UAMC1110 at a 10 mg/kg concentration was co-injected with [^64^Cu]Cu-CB-TE2A-(FAPI-04)_2_ for blockade.

## 3. Results

### 3.1. Structural Design and Synthesis of CB-TE2A-FAPI-04 and CB-TE2A-(FAPI-04)_2_

**Scheme 1:**
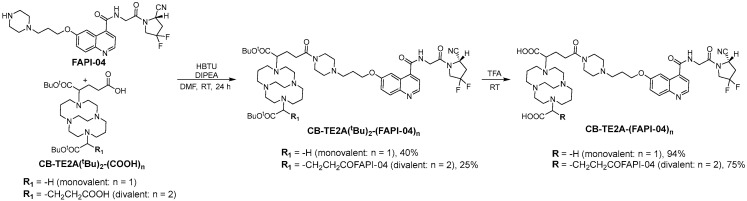
Synthetic route to monovalent and divalent FAPI-04 conjugates. Reagents and reaction condition for monovalent conjugate: FAPI-04 (1.2 equiv.), CB-TE2A(*^t^*Bu)_2_-COOH (1 equiv.), HBTU (1 equiv.), DIPEA (5 equiv.), 24 h; TFA, 6 h. Reagents and reaction condition for divalent conjugate: FAPI-04 (3.5 equiv.), CB-TE2A(*^t^*Bu)_2_-(COOH)_2_ (1 equiv.), HBTU (2 equiv.), DIPEA (10 equiv.), 24 h; TFA, 3 h.

The desired monovalent and divalent FAPI-04 conjugates [CB-TE2A-(FAPI-04)_n_, n = 1 or 2] were prepared following a synthetic scheme presented in Scheme 1. Prior to the conjugation, bicyclic CB-TE2A(*^t^*Bu)_2_-COOH and CB-TE2A(*^t^*Bu)_2_-(COOH)_2_ derivatives were synthesized according to our reported procedure [27]. FAPI-04 ligand was synthesized following a known synthetic route reported in the literature(Scheme S1) [12]. FAPI-04 in crude form obtained from the deprotection of Boc-FAPI-04 was coupled with CB-TE2A(*^t^*Bu)_2_-COOH and CB-TE2A(*^t^*Bu)_2_-(COOH)_2_ in the presence of HBTU to give desired *^t^*Bu-protected monovalent CB-TE2A(*^t^*Bu)_2_-FAPI-04 and divalent CB-TE2A(*^t^*Bu)_2_-(FAPI-04)_2_ conjugates in moderate yields (Scheme 1). Finally, *^t^*Bu- groups were removed using TFA to obtain the desired precursor conjugates CB-TE2A-FAPI-04 and CB-TE2A-(FAPI-04)_2_ in 94% and 75% yields, respectively. Of note, the reaction time for the synthesis of CB-TE2A-(FAPI-04)_2_ must be kept under 3 h. Otherwise, hydration of cyanide (-CN) to amide (-CONH_2_) would occur in the presence of TFA. Surprisingly, such cyanide hydration was not observed in the synthesis of the monovalent conjugate, CB-TE2A-FAPI-04, even after 6 h of reaction.

### 3.2. Radiolabeling of CB-TE2A-FAPI-04 and CB-TE2A-(FAPI-04)_2_ with ^64^Cu

Radiolabeling of the monovalent and divalent conjugates with ^64^Cu was successful with non-decay corrected radiochemical yields (RCY) of ~60% (Scheme S2). The radiochemical purity of both [^64^Cu]Cu-CB-TE2A-FAPI-04 and [^64^Cu]Cu-CB-TE2A-(FAPI-04)_2_ was >96% (Figures S7 and S8) as determined by radio-HPLC. The calculated molar activity at the end of radiosynthesis was 37 ± 3.1 GBq/μmol for [^64^Cu]Cu-CB-TE2A-FAPI-04 (n = 3) and 52 ± 4.7 GBq/μmol for [^64^Cu]Cu-CB-TE2A-(FAPI-04)_2_ (n = 4). In a biphasic octanol and PBS mixture, the partition coefficients were determined to be −1.6 and −1.2 for [^64^Cu]Cu-CB-TE2A-FAPI-04 and [^64^Cu]Cu-CB-TE2A-(FAPI-04)_2_, respectively, reflecting their hydrophilic nature.

### 3.3. In Vitro Assays

#### 3.3.1. FAP Expression in Tumor Cell Line and Tissues

The RENCA-FAP cells were validated with positive FAP immunofluorescence stain (Figure S9A–C), while RENCA cells showed no FAP immunofluorescence (Figure S9D–F). Similarly, the FAP expression in RENCA-FAP tumors was confirmed with immunofluorescence staining. In addition, the FAP expression in XP-185 tumorgrafts was confirmed with Western blot (Figure S10A) and immunohistochemistry (IHC) (Figure S10B) staining using an anti-FAP-α antibody.

#### 3.3.2. Stability of [^64^Cu]Cu-CB-TE2A-FAPI-04 and [^64^Cu]Cu-CB-TE2A-(FAPI-04)_2_ in Human Serum

The in vitro stability of [^64^Cu]Cu-CB-TE2A-FAPI-04 and [^64^Cu]Cu-CB-TE2A-(FAPI-04)_2_ was evaluated in human serum by radio-HPLC. The assays demonstrated no ^64^Cu dissociation from either of the conjugates nor decomposition of the radiolabeled conjugates out to 24 h of incubation, indicative of the expected robustness of the radiotracers (Figure S11).

#### 3.3.3. FAP-Specific Cell Uptake and FAP-Mediated Internalization

**Figure 2:**
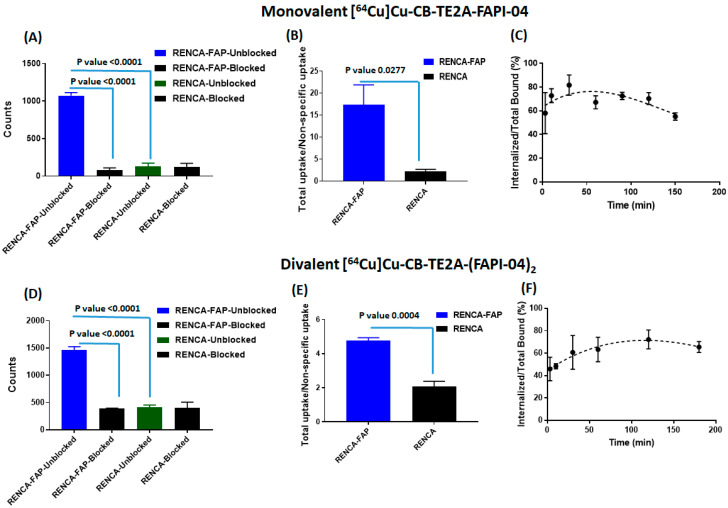
Cell-based FAP-binding affinity and internalization assays of [^64^Cu]Cu-CB-TE2A-FAPI-04 (upper panel) and [^64^Cu]Cu-CB-TE2A-(FAPI-04)_2_ (lower panel). (**A**,**D**): FAP-specific binding assay using UAMC1110 as blockade; (**B**,**E**): Calculated ratios of total uptake vs. nonspecific uptake; (**C**,**F**): time-dependent internalization. Cell lines: RENCA-FAP (FAP^+^) and RENCA (FAP^−^).

Cell-based FAP-binding affinities of [^64^Cu]Cu-CB-TE2A-FAPI-04 and [^64^Cu]Cu-CB-TE2A-(FAPI-04)_2_ were assessed in RENCA-FAP and RENCA cells (Figure 2A,D). The total cell uptake vs. nonspecific uptake ratio of [^64^Cu]Cu-CB-TE2A-FAPI-04 was ~17.38 in RENCA-FAP cells and only ~2.19 in RENCA cells (Figure 2B). However, the ratios were reduced to ~4.77 and ~2.09, respectively, for [^64^Cu]Cu-CB-TE2A-(FAPI-04)_2_ (Figure 2E). In addition, blockade using 1 mM of UAMC1110 resulted in a ~14-fold and ~4-fold reduction in FAP binding for [^64^Cu]Cu-CB-TE2A-FAPI-04 and [^64^Cu]Cu-CB-TE2A-(FAPI-04)_2_, respectively, in RENCA-FAP cells. On the other hand, their accumulation in RENCA cells was unaltered in the presence of UAMC1110 (Figure 2A,D). Furthermore, both [^64^Cu]Cu-CB-TE2A-FAPI-04 and [^64^Cu]Cu-CB-TE2A-(FAPI-04)_2_ showed time-dependent internalization in a RENCA-FAP cell. The monovalent [^64^Cu]Cu-CB-TE2A-FAPI-04 exhibited rapid internalization. By contrast, the divalent [^64^Cu]Cu-CB-TE2A-(FAPI-04)_2_ displayed a different internalization profile. After an initial gradual increase (30–60 min) (Figure 2C,F), it reached maximum internalization at a much later time (90–120 min). Taken together, both [^64^Cu]Cu-CB-TE2A-FAPI-04 and [^64^Cu]Cu-CB-TE2A-(FAPI-04)_2_ exhibited FAP-specific uptake and time-dependent internalization, which are desired features for cancer theranostic agents.

### 3.4. In Vivo PET/CT Imaging of [^64^Cu]Cu-CB-TE2A-FAPI-04 and [^64^Cu]Cu-CB-TE2A-(FAPI-04)_2_

**Figure 3:**
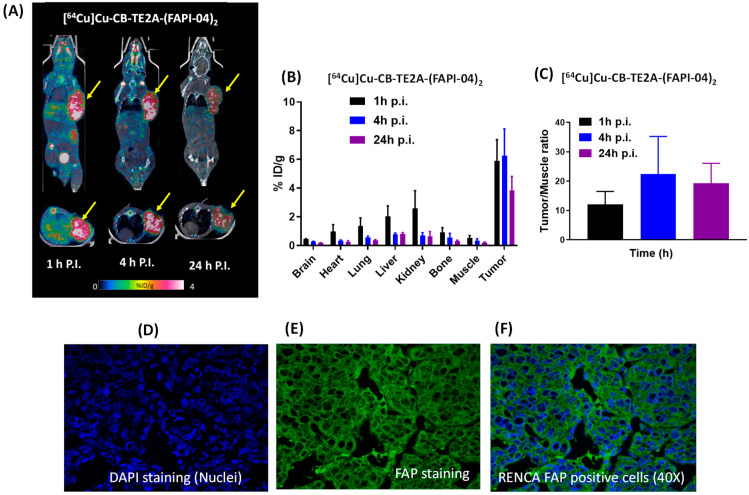
(**A**): Representative PET/CT images of [^64^Cu]Cu-CB-TE2A-(FAPI-04)_2_ in mice bearing RENCA-FAP (FAP^+^) tumors (yellow arrows indicate the tumors) at different time points. (**B**): Biodistribution of [^64^Cu]Cu-CB-TE2A-(FAPI-04)_2_ by quantitative imaging data analysis in the mice. Data presented as average %ID/g ± standard deviation (s.d.) (n = 3). (**C**): Tumor/muscle ratio at different time points. (**D**–**F**): Immunofluorescence staining assays of excised RENCA-FAP tumor tissues post imaging. The nuclei were stained with DAPI (**D**) and tumor cells with anti-FAP antibody (**E**). (**F**): Overlay of the FAP and DAPI staining.

First, a proof-of-concept PET/CT imaging study was performed with the divalent [^64^Cu]Cu-CB-TE2A-(FAPI-04)_2_ in BALB/c mice subcutaneously bearing RENCA-FAP (FAP^+^) tumors. Demonstrated in Figure 3A, [^64^Cu]Cu-CB-TE2A-(FAPI-04)_2_ clearly visualized the RENCA-FAP tumors with high contrast. Quantitative imaging data analysis (Figure 3B) revealed that the tumor uptake of [^64^Cu]Cu-CB-TE2A-(FAPI-04)_2_ was 6.25 ± 1.87% ID/g at 4 h p.i. By contrast, its uptake in the muscle was only 0.33 ± 0.13% ID/g at 4 h p.i. and maintained at a similar level out to 24 h p.i. The tumor-to-muscle ratios were 12.04, 22.49, and 19.32 at 1 h, 4 h, and 24 h p.i., respectively (Figure 3C). Furthermore, [^64^Cu]Cu-CB-TE2A-(FAPI-04)_2_ showed low-to-moderate uptake in other non-target tissues (e.g., kidneys: 0.78 ± 0.18% ID/g; liver: 0.78 ± 0.08% ID/g; brain: 0.27 ± 0.02% ID/g; heart: 0.34 ± 0.04% ID/g; lungs: 0.57 ± 0.08% ID/g; and bone: 0.56 ± 0.29% ID/g, 4 h p.i.). Immunofluorescence staining of the excised tumor tissues post imaging validated the expression of FAP in the tumors (Figure 3D–F).

**Figure 4:**
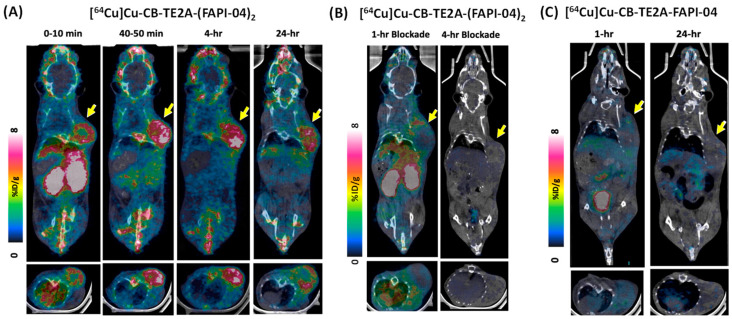
(**A**): Representative PET/CT images of XP-185 tumorgrafts in SCID/NOD mice (n = 4) with divalent [^64^Cu]Cu-CB-TE2A-(FAPI-04)_2_. (**B**): Images obtained with co-injection of UAMC1110 with divalent [^64^Cu]Cu-CB-TE2A-(FAPI-04)_2_. (**C**): Representative PET/CT images of XP-185 tumorgrafts in SCID/NOD mice (n = 4) with monovalent [^64^Cu]Cu-CB-TE2A-FAPI-04. Tumors are indicated with yellow arrows.

**Figure 5:**
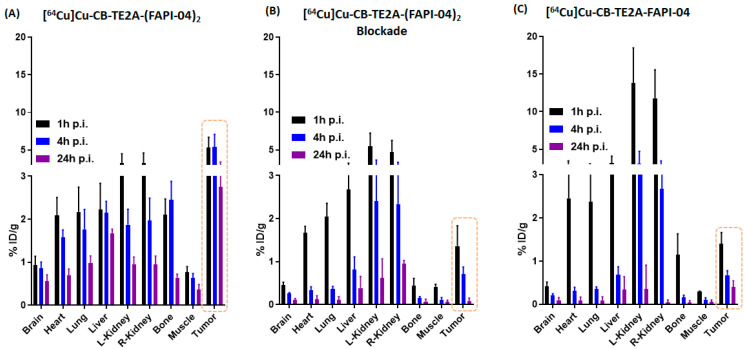
Quantitative analysis of PET signals in tissues of interest in SCID/NOD mice bearing XP-185 tumorgrafts (n = 4). (**A**): [^64^Cu]Cu-CB-TE2A-(FAPI-04)_2_. (**B**): [^64^Cu]Cu-CB-TE2A-(FAPI-04)_2_ co-injected with UAMC1110 (tumor blocked vs. tumor unblocked at 4 h p.i., *p* = 0.0093.). (**C**): [^64^Cu]Cu-CB-TE2A-FAPI-04. Tumors are indicated with orange dotted boxes.

Furthermore, a comparative imaging study was carried out between divalent [^64^Cu]Cu-CB-TE2A-(FAPI-04)_2_ and monovalent [^64^Cu]Cu-CB-TE2A-FAPI-04 in a clinically relevant mouse model, which is a renal cell carcinoma tumorgraft (XP-185) established in SCID/NOD mice [39]. While the tumors could be visualized by both [^64^Cu]Cu-CB-TE2A-(FAPI-04)_2_ and [^64^Cu]Cu-CB-TE2A-FAPI-04 (Figure 4), the divalent one exhibited markedly higher tumor uptake (3–15 times) than the monovalent throughout the scans. The tumor uptake of the divalent one peaked at 4 h p.i. (5.3 ± 1.7% ID/g). By contrast, the monovalent one only showed moderate tumor uptake at 4 h p.i. (0.67 ± 0.1 ID/g) (Figure 5 and Table 1). Impressively, both the divalent [^64^Cu]Cu-CB-TE2A-(FAPI-04)_2_ and monovalent [^64^Cu]Cu-CB-TE2A-FAPI-04 exhibited a rapid and effective clearance profile from non-target tissues through kidneys, which can be attributed to their hydrophilic nature and in vivo stability [40]. Of note, ^64^Cu dislocated from the conjugates would end up in the liver. As anticipated, the divalent [^64^Cu]Cu-CB-TE2A-(FAPI-04)_2_ showed significantly higher tumor uptake retention (2.5 ± 0.4 ID/g at 24 h p.i.) than its monovalent counterpart (0.16 ± 0.01 at 24 h p.i.) throughout the imaging study (*p* < 0.005), which is the desired property for an efficacious radiotheranostic agent.

To assess the FAP-targeted imaging specificity, a blocking study was conducted by co-injecting 100 µg of UAMC1110. As shown in Figure 4B and Figure 5B, the tumor uptake of [^64^Cu]Cu-CB-TE2A-(FAPI-04)_2_ was reduced by 8-fold with UAMC1110 blockade at 4 h p.i. This demonstrates the desired imaging specificity of FAP. Of note, the delayed renal clearance of [^64^Cu]Cu-CB-TE2A-(FAPI-04)_2_ might result from the presence of an excessive amount of its competitive FAP inhibitor, UAMC1110, in blood circulation [41].

Both [^64^Cu]Cu-CB-TE2A-(FAPI-04)_2_ and [^64^Cu]Cu-CB-TE2A-FAPI-04 are specific radiotracers for PET imaging of FAP expression. For future translational study planning, we will further evaluate the human radiation dosimetry of the agents, particularly for the radioligand therapy with their ^67^Cu-labeled counterparts to maximize their treatment efficacies while minimizing the side-effects, which might result from off-target uptake.

## 4. Discussion

With the recent success of FAP-targeted imaging agents, targeting the ubiquitous presence of FAP in the TME has become a practical approach to design and develop radiotheranostic agents for efficacious pan-tumor diagnosis and treatment [7]. In this regard, the current strategy is to improve the therapeutic index with an imaging-guided precision treatment that targets the upregulated expression of FAP in solid tumors. In terms of developing theranostic radiopharmaceuticals, the challenge is mainly defined by the availability of a pair of imaging and therapeutic radionuclides, which matches the physiochemical and biological properties of the chosen targeting molecule. Given the established role of FAPI-04 in FAP-targeted agent development, we set out to develop FAP-targeted radiotheranostic agents using FAPI-04 as the targeting moiety and the ideal pair of ^64^Cu and ^67^Cu for imaging and radiotherapy, respectively. It is noteworthy that both ^64^Cu and ^67^Cu have become available in the community for novel copper radiopharmaceutical development.

The pair of ^64^Cu and ^67^Cu offers substantial benefits over other theranostic radionuclide pairs such as ^68^Ga/^177^Lu [25]. First, the extended physical half-life of ^64^Cu (t_1/2_ = 12.7 h) allows for centralized production and nationwide transportation, which overcomes the constraint by the short half-life of ^68^Ga (t_1/2_ = 68 min). In addition, the half-life difference between ^64^Cu and ^67^Cu is much smaller than that between ^68^Ga and ^177^Lu. This will widen the imaging window that ^68^Ga could offer and permit the use of ^64^Cu to assess the radiation dosimetry of ^67^Cu through a chemically identical pair of copper radiopharmaceuticals. Furthermore, the abundant γ-photons from ^67^Cu can also be used for SPECT imaging in places where SPECT scanners are available [42]. Importantly, with a half-life shorter than that of ^177^Lu (t_1/2_ = 6.7 d), ^67^Cu is better suited for theranostic radiopharmaceutical development from small organic molecules or peptides. However, small molecules suffer limited tumor uptake retention resulting from rapid in vivo clearance, which hampers the desired therapeutic application.

In this work, we present a radiotheranostic design represented by [^64/67^Cu]Cu-CB-TE2A-(FAPI-04)_n_ (n = 1 or 2) to overcome the inherent challenge imposed by the rapid in vivo kinetics of small molecules. Our strategy is to present multiple copies of FAPI-04 on an ideal chelator scaffold we have reported for copper radiopharmaceuticals. The goal is to enhance the desired high tumor retention for efficacious cancer treatment while maintaining the rapid clearance of small molecules from non-target organs and in vivo stability of the copper radiopharmaceuticals to minimize the unwanted side effects. For the practicality of our methodology, we designed a modular strategy for the synthesis of the monovalent CB-TE2A-FAPI-04 and divalent CB-TE2A-(FAPI-04)_2_ conjugates using the CB-TE2A BFS through an aliphatic linker.

The radiolabeling of both conjugates with ^64^Cu was straightforward with high radiochemical yields to afford [^64^Cu]Cu-CB-TE2A-FAPI-04 and [^64^Cu]Cu-CB-TE2A-(FAPI-04)_2_ conjugates in high radiochemical purity. Given the pilot nature of this work, radiotherapy studies with their ^67^Cu counterparts are not performed. In addition, the radiolabeling conditions were not optimized to maximize the molar activity of the radiotheranostic agents for the in vitro and in vivo studies presented herein. Because both agents are FAP-targeted, we will improve their molar activity to a level as high as achievable in future studies. As anticipated, both [^64^Cu]Cu-CB-TE2A-FAPI-04 and [^64^Cu]Cu-CB-TE2A-(FAPI-04)_2_ demonstrated the anticipated binding affinity and specificity to FAP in addition to their high in vitro stability, which are better than or similar to the reported agents (Table S1). Of note, because different xenograft models were used in the reported studies, the tumor uptake levels of the agents in Table S1 are not comparable. However, by directly comparing the divalent [^64^Cu]Cu-CB-TE2A-(FAPI-04)_2_ with the monovalent [^64^Cu]Cu-CB-TE2A-FAPI-04, we observed significantly increased uptake and prolonged retention of the divalent one in two tumor mouse models, one of which is a clinically relevant patient-derived xenograft model. This mainly reflects the validity of our multivalent design strategy [43]. However, whether both FAPI-04 moieties can effectively bind to the surface FAP sites simultaneously remains to be further addressed by varying the linker’s length, lipophilicity, and flexibility. It is noteworthy that the binding cavity of FAP is reported to be around 300–500 Å^3^, in which the divalent [^64^Cu]Cu-CB-TE2A-(FAPI-04)_2_ with increased molecular size might find a better fitting than its monovalent counterpart [44,45]. This warrants future studies on the molecular mechanism of the observed tumor uptake and retention differences between the divalent and monovalent constructs.

## 5. Conclusions

As FAPI-targeted theranostics continue to evolve, they hold a significant potential to revolutionize precision treatment strategies for solid tumors. Toward this goal, we have designed and developed a BFS-based modular synthetic approach to generate multivalent FAP-targeted radiotheranostic agents from a small-molecule-targeting moiety. Pilot biological studies of two model conjugates labeled with ^64^Cu demonstrated the success of this strategy for enhancing tumor uptake and retention, which presents a convincing premise for future development of efficacious cancer treatments with ^67^Cu. Furthermore, the versatile chemical platform presented in this work offers advantages over antibody drug conjugates in terms of in vivo kinetics, solid tumor penetration, definitive chemical structure, and adaptability for other small-molecule-based theranostic agent development.

## Figures and Tables

**Table 1:** Comparative tumor uptake of divalent [^64^Cu]Cu-CB-TE2A-(FAPI-04)_2_ with monovalent [^64^Cu]Cu-CB-TE2A-FAPI-04 as measured by PET imaging quantification. Data are presented as %ID/g ± s.d. (n = 4); NP = Not performed.

	1 h	4 h	24 h	4 h Blockade
[^64^Cu]Cu-CB-TE2A-(FAPI-04)_2_	5.3 ± 1.4	5.3 ± 1.7	2.5 ± 0.4	0.67 ± 0.12
[^64^Cu]Cu-CB-TE2A-FAPI-04	1.4 ± 0.5	0.67 ± 0.1	0.16 ± 0.01	NP

## Data Availability

The data presented in this study are available in this article and Supplementary Materials.

## Supplementary Materials

The following supporting information can be downloaded at: https://www.mdpi.com/article/10.3390/cancers16244180/s1. It includes Scheme S1: Synthetic route to Boc-FAPI-04; Scheme S2: Radiolabeling reaction of monovalent and divalent conjugates with [^64^Cu]CuCl_2_ to produce [^64^Cu]Cu-CB-TE2A-FAPI-04 and [^64^Cu]Cu-CB-TE2A-(FAPI-04)_2_; Synthetic procedure of compound 3 to 9; Figure S1: HPLC chromatogram of Boc-FAPI-04; Figure S2: MS (ESI) of Boc-FAPI-04; Figure S3: HPLC chromatogram of CB-TE2A-FAPI-04; Figure S4: MS (ESI) of CB-TE2A-FAPI-04; Figure S5: HPLC chromatogram of CB-TE2A-(FAPI-04)_2_; Figure S6: MS (ESI) of CB-TE2A-(FAPI-04)_2_; Figure S7: HPLC chromatograms of [^64^Cu]Cu-CB-TE2A-FAPI-04; Figure S8: HPLC chromatograms of [^64^Cu]Cu-CB-TE2A-(FAPI-04)_2_; Figure S9: Top panel for RENCA-FAP (FAP^+^) cell and bottom panel for RENCA (FAP^−^) cell; Figure S10: (A) Western blot of FAP on XP185, (B) Immunohistochemistry staining of XP-185 tumor xenograft using FAP-α-antibody; Figure S11: In vitro stability of (A) [^64^Cu]Cu-CB-TE2A-FAPI-04 and (B) [^64^Cu]Cu-CB-TE2A-(FAPI-04)_2_ in human serum; Table S1: Summary of [^64^Cu]Cu-CB-TE2A-FAPI-04 and [^64^Cu]Cu-CB-TE2A-(FAPI-04)_2_ vs. Reported FAP-targeted agents. References [18,46,47,48,49,50,51] are cited in the Supplementary Materials.
