# Supplementary material for: Monovalent and Divalent Designs of Copper Radiotheranostics Targeting Fibroblast Activation Protein in Cancer
Source: Cancers (Basel). 2024 Dec 15;16(24):4180. doi: 10.3390/cancers16244180 (PMC11675001; doi:10.3390/cancers16244180)
Supplement: Supplementary file 1 [file cancers-16-04180-s001.zip › cancers-3333388-supplementary.pdf]

Supplementary Information

Monovalent and Divalent Designs of Copper Radiotheranostics Targeting Fibroblast Activation Protein in Cancer

Pawan Thapa [1,†], Sashi Debnath [1,†], Anjan Bedi [1], Madhuri Parashar [2], Paulina Gonzalez [1], Joshua Reus [2], Hans Hammers [2,3] and Xiankai Sun [1,3,4,*]

[1] Department of Radiology, University of Texas Southwestern Medical Center, Dallas, TX 75390, USA; ppawann86@gmail.com (P.T.), sashi.debnath@utsouthwestern.edu (S.D.), anjan.bedi@utsouthwestern.edu (A.B.), paulina.gonzalez@utsouthwestern.edu (P.G.)
[2] Department of Internal Medicine, University of Texas Southwestern Medical Center, Dallas, TX 75390, USA; madhurriparashar@gmail.com (M.P.), joshua.reus@utsouthwestern.edu (J.R.); hans.hammers@utsouthwestern.edu (H.H.)
[3] Kidney Cancer Program, University of Texas Southwestern Medical Center, Dallas, TX 75390, USA
[4] Advanced Imaging Research Center, University of Texas Southwestern Medical Center, Dallas, TX 75390, USA
[*] Correspondence: xiankai.sun@utsouthwestern.edu
[†] This authors contributed equally to this work.

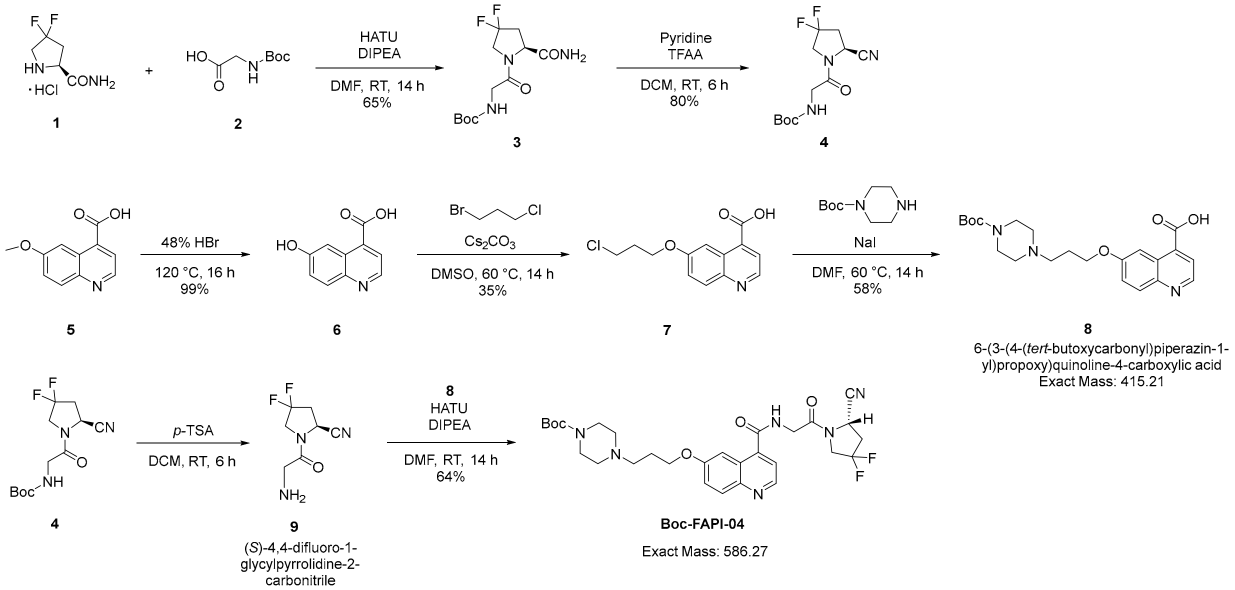

Scheme S1. Synthetic route to Boc-FAPI-04.

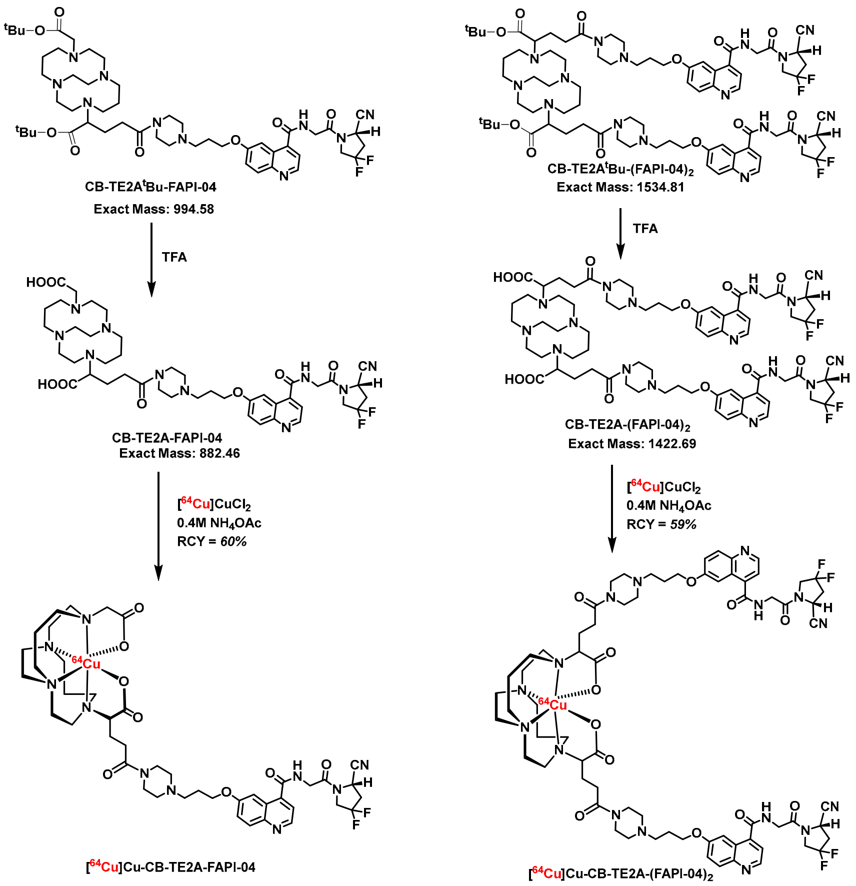

Scheme S2. Radiolabeling reaction of monovalent and divalent conjugates with [^{64}Cu]$CuCl_2$ to produce [^{64}Cu]Cu-CB-TE2A-FAPI-04 and [^{64}Cu]Cu-CB-TE2A-(FAPI-04)$_2$.

Synthesis of (*S*)-*tert*-butyl (2-(2-carbamoyl-4,4-difluoropyrrolidin-1-yl)-2-oxoethyl)carbamate (3) :
A solution of 2-((*tert*-butoxycarbonyl)amino)acetic acid **2** (103.3 mg, 0.59 mmol) in 0.5 mL DMF was added to the mixture of (S)-4,4-difluoropyrrolidine-2-carboxamide hydrogen chloride **1** (100.8 mg, 0.54 mmol), HATU (225.0 mg, 0.59 mmol) and DIPEA (282.0 μL, 1.62 mmol) in 0.5 mL DMF. The resulting mixture was stirred at room temperature for 14 h. The reaction mixture was diluted with 1.0 mL water and purified by reverse-phase HPLC (10% acetonitrile/90% H_2O to 60% acetonitrile/40% H_2O in 25 min; all solvents contained 0.1 percent TFA) to afford 108.1 mg (65%) desired product **3** as white solid. MS (ESI) *m*/*z* calcd for $C_{12}H_{19}F_2N_3O_4$: 307.13; found 330.11 $[M+Na]^+$.

Synthesis of (*S*)-*tert*-butyl (2-(2-cyano-4,4-difluoropyrrolidin-1-yl)-2-oxoethyl)carbamate (4):
Compound **3** (30.8 mg, 0.10 mmol) was dissolved in anhydrous dichloromethane (DCM) at room temperature. Pyridine (8.0 μL, 0.10 mmol) and trifluoroacetic anhydride (TFAA) (14.0 μL, 0.10 mmol) were added successively. After stirring the solution for 2 h at room temperature, another portion of pyridine and TFAA were added. The solution was further stirred for 4 h at room temperature. The solvent was removed under reduced pressure and the crude product was dissolved in 1 mL acetonitrile. The crude product solution was injected into reverse-phase HPLC for purification to afford 23.1 mg (80%) of the desired product **4** as a white solid. MS (ESI) *m*/*z* calcd for $C_{12}H_{17}F_2N_3O_3$: 289.12; found 290.13 $[M+H]^+$, 312.11 $[M+Na]^+$.

Synthesis of 6-hydroxyquinoline-4-carboxylic acid (6):

A mixture of 6-methoxyquinoline-4-carboxylic acid (**5**) (101.6 mg, 0.50 mmol) and 2.5 mL 48% HBr solution was heated at 120 °C for 16 h. Yellow crystals were formed after the solution was cooled to room temperature. The solid crystals were collected through filtration and were thoroughly washed with acetone followed by diethyl ether to afford 93.6 mg (99%) of the desired product **6**.

Synthesis of 6-(3-chloropropoxy)quinoline-4-carboxylic acid (7):

A mixture of compound **6** (37.8 mg, 0.20 mmol), cesium carbonate (325 mg, 1.00 mmol) and 1-bromo-3-chloropropane (126 mg, 0.80 mmol) in 1.0 mL dimethyl sulfoxide (DMSO) was stirred at 60 °C for 14 h. After completion of the reaction, the solution was cooled to room temperature and diluted with 1.0 mL water followed by 1.0 mL acetonitrile. The mixture was added with 0.5 mL 6 M sodium hydroxide (NaOH) and stirred for 2 hours at room temperature. The undissolved solids were filtered out and the crude product in the filtrate was purified with reverse-phase HPLC to afford 19.0 mg (35%) of the desired product **7** as a pale-yellow solid. MS (ESI) *m/z* calcd for $C_{13}H_{12}ClNO_3$: 265.05; found 266.06 $[M+H]^+$.

Synthesis of 6-(3-(4-(*tert*-butoxycarbonyl)piperazin-1-yl)propoxy)quinoline-4-carboxylic acid (8):

In a pressure relief reaction vial, compound **7** (26.5 mg, 0.10 mmol), sodium iodide (100.0 mg, 0.67 mmol) and *tert*-butyl piperazine-1-carboxylate (100 mg, 0.54 mmol) were dissolved in 0.5 mL DMF. The reaction mixture was stirred at 60 °C for 14 h. The mixture was cooled to room temperature and diluted with 0.5 mL water and 0.5 mL acetonitrile. The crude product was purified by reverse-phase HPLC to afford 24.1 mg (58%) of the desired product **8** as a white solid. MS (ESI) *m/z* calcd for $C_{22}H_{29}N_3O_5$: 415.21; found 416.22 $[M+H]^+$.

Synthesis of (*S*)-4,4-difluoro-1-glycylpyrrolidine-2-carbonitrile (9):

In a pressure relief reaction vial, compound **8** (21 mg, 0.05 mmol) and *p*-toluenesulfonic acid (*p*-TSA) (12 mg, 0.07 mmol) were dissolved in 0.5 mL DCM. The reaction mixture was stirred at room temperature for 6 h. The mixture was evaporated and analyzed for product formation. The crude product obtained was used for the subsequent reaction without further purification. MS (ESI) *m/z* calcd for $C_{22}H_{29}N_3O_5$: 189.07; found 190.07 $[M+H]^+$.

Characterization

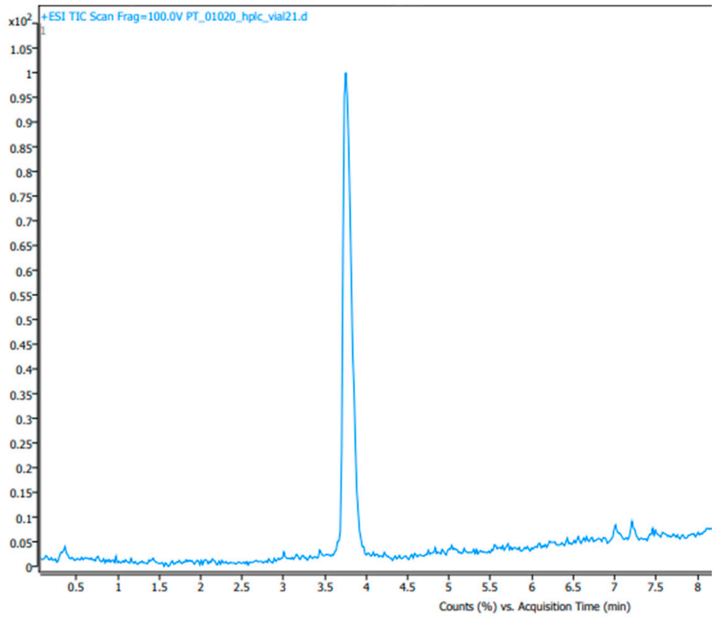

Figure S1. HPLC chromatogram of Boc-FAPI-04.

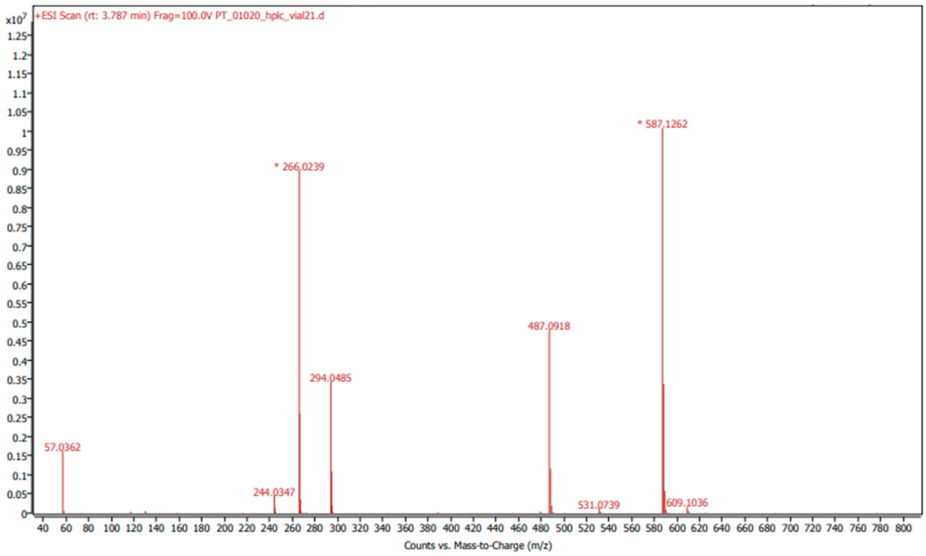

Figure S2. MS (ESI) of Boc-FAPI-04.

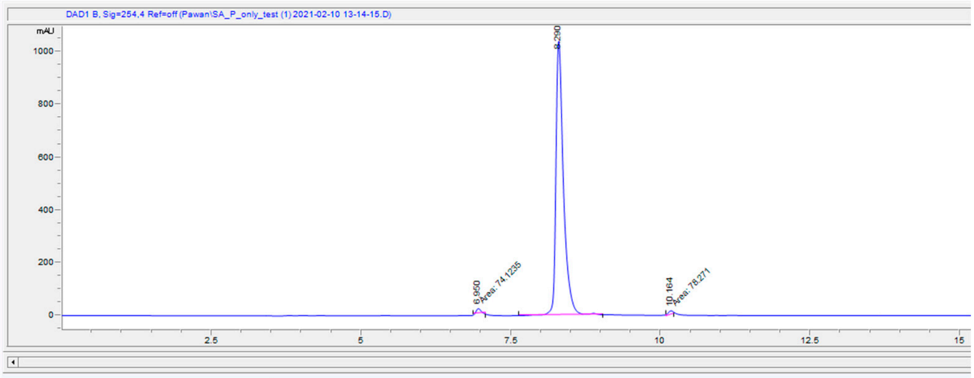

#	Time	Type	Area	Height	Width	Area%	Symmetry
1	6.95	MM	74.1	16.7	0.0742	0.793	0.863
2	8.29	BV R	9191.2	1043.4	0.1302	98.369	0.56
3	10.164	MM	78.3	14.5	0.0901	0.838	1.388

Figure S3. HPLC chromatogram of CB-TE2A-FAPI-04.

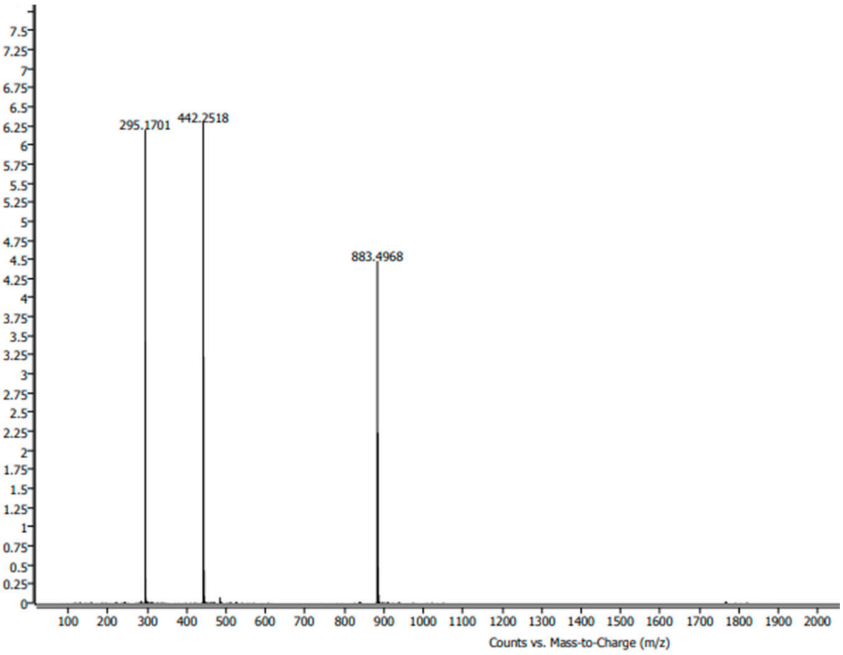

Figure S4. MS (ESI) of CB-TE2A-FAPI-04.

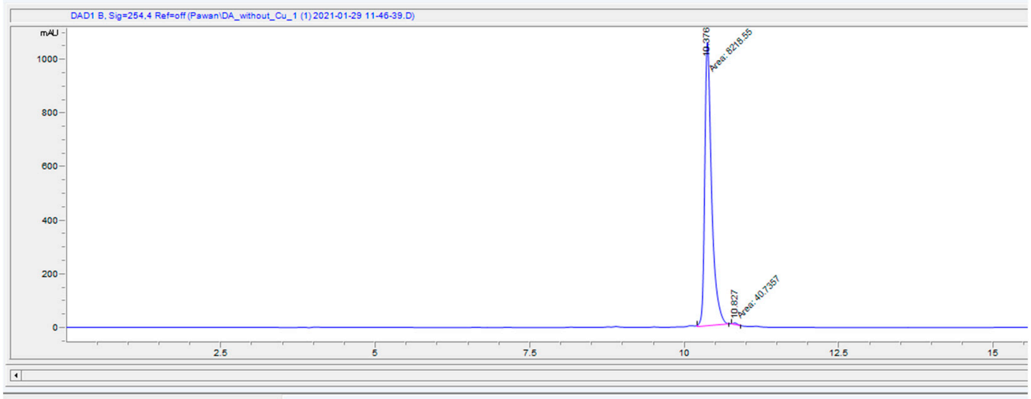

#	Time	Type	Area	Height	Width	Area%	Symmetry
1	10.376	MM	8218.6	1060.7	0.1291	99.507	0.601
2	10.827	MM	40.7	7.2	0.0942	0.493	0.555

Figure S5. HPLC chromatogram of CB-TE2A-(FAPI-04)$_2$.

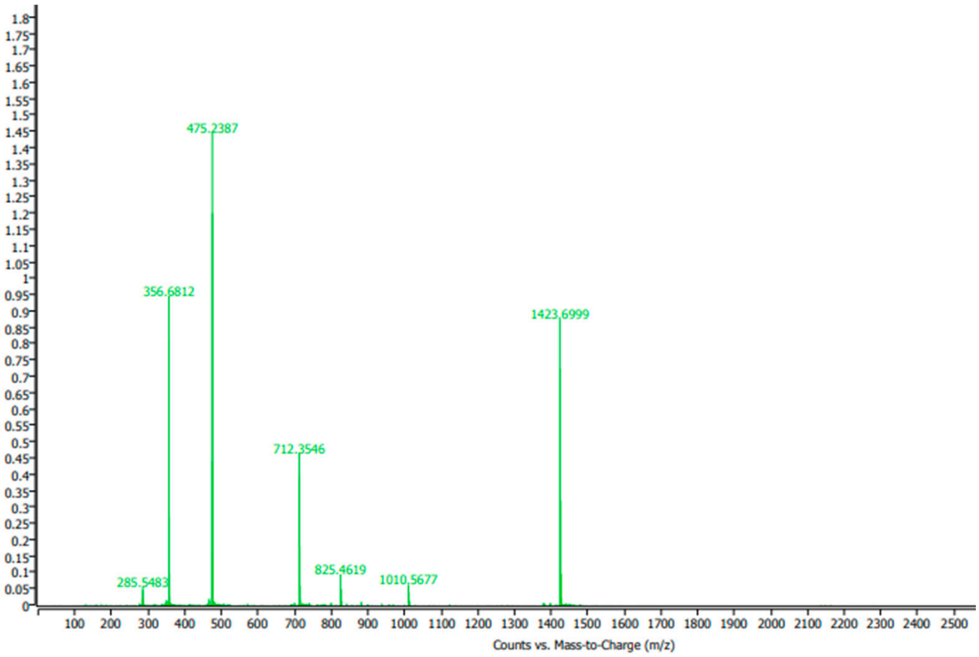

Figure S6. MS (ESI) of CB-TE2A-(FAPI-04)$_2$.

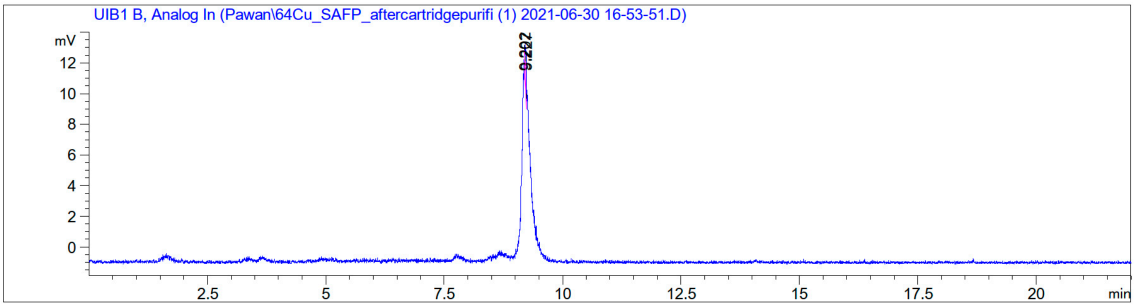

Figure S7. HPLC chromatograms of [^{64}Cu]Cu-CB-TE2A-FAPI-04.

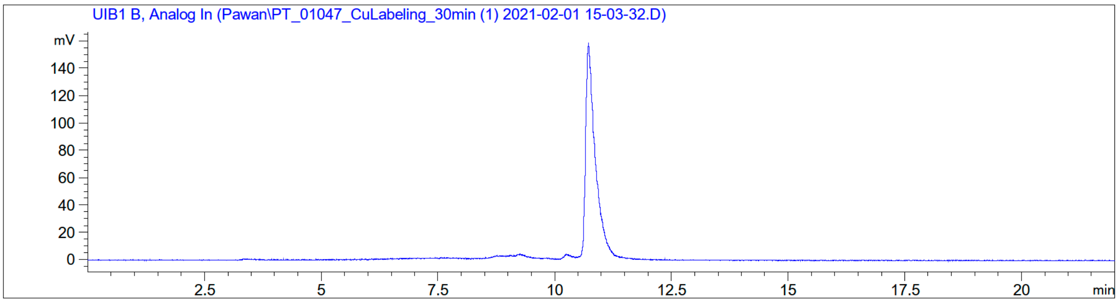

Figure S8. HPLC chromatograms of [^{64}Cu]Cu-CB-TE2A-(FAPI-04)$_2$.

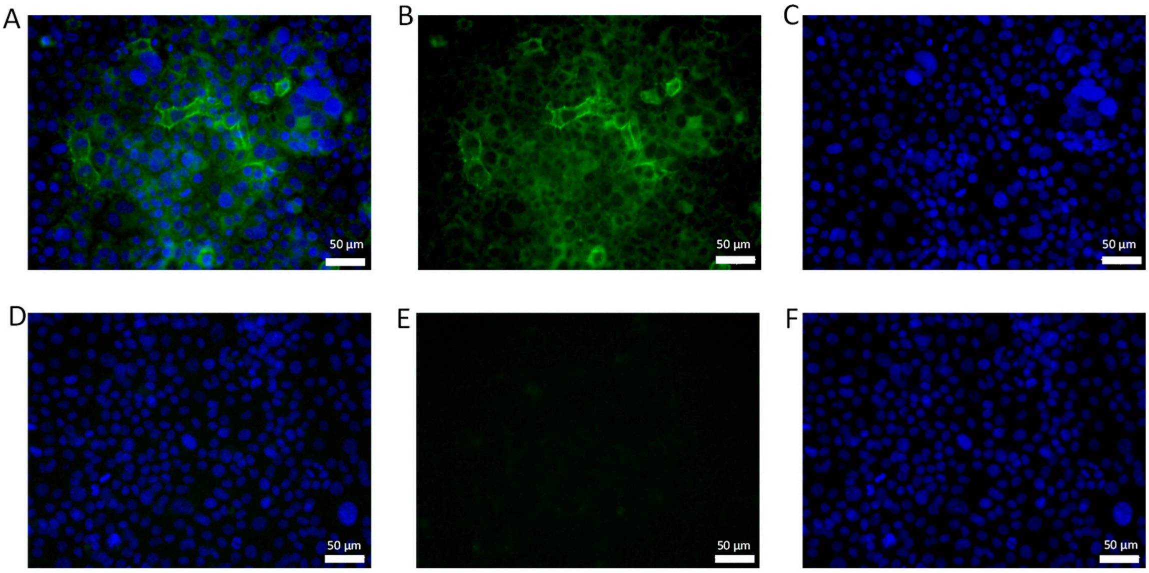

Figure S9. Top panel for RENCA-FAP (FAP$^+$) cell and bottom panel for RENCA (FAP$^-$) cell. FAP cell line validation through immunofluorescence staining. The RENCA-FAP (FAP$^+$) cell (A) FAP and DAPI both, (B) FAP only, (C) DAPI only, and the FAP negative RENCA (FAP$^-$) cell (D) FAP and DAPI both, (E) FAP only, (F) DAPI only.

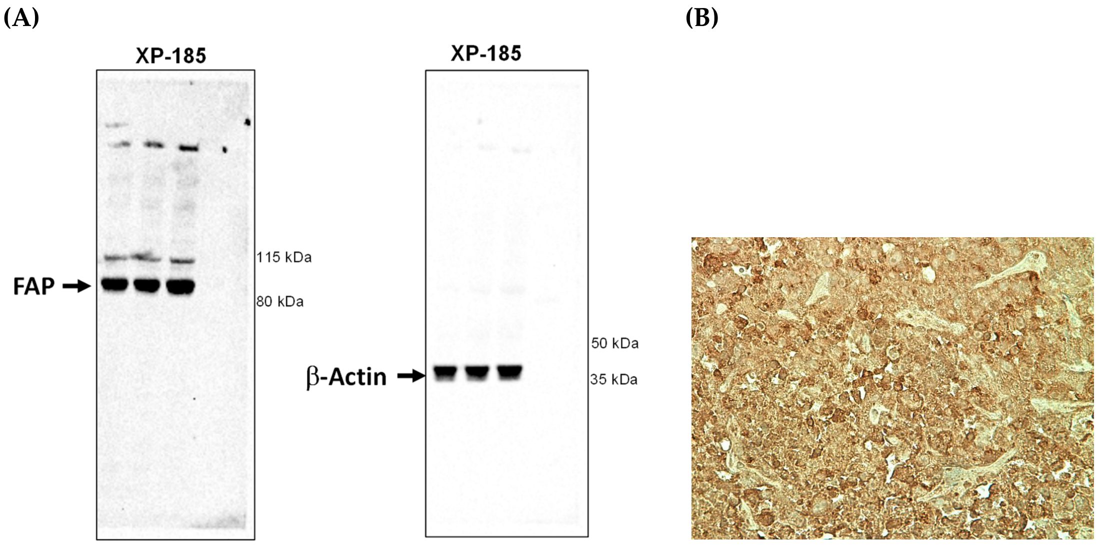

Figure S10. (A) Western blot of FAP on XP185, (B) Immunohistochemistry staining of XP-185 tumor xenograft using FAP-α-antibody.

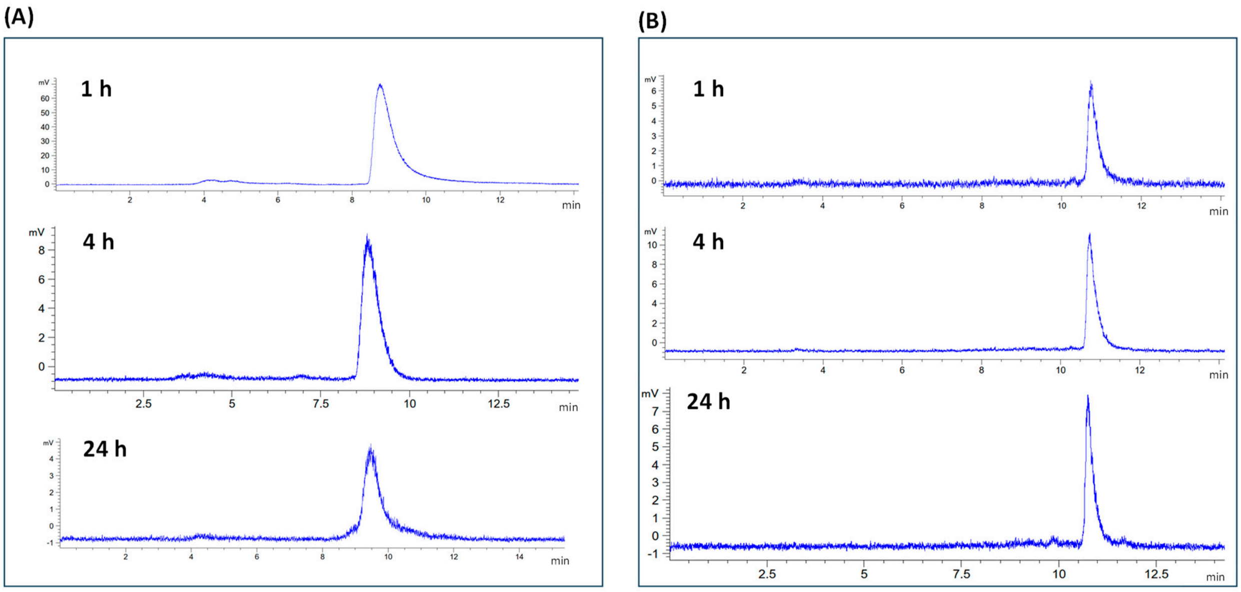

Figure S11. In vitro stability of (A) [^{64}Cu]Cu-CB-TE2A-FAPI-04 and (B) [^{64}Cu]Cu-CB-TE2A-(FAPI-04)$_2$ in human serum at 1h, 4h and 24h post incubation.

Table S1. Summary of [^{64}Cu]Cu-CB-TE2A-FAPI-04 and [^{64}Cu]Cu-CB-TE2A-(FAPI-04)$_2$ vs. Reported PET agents.

PET Tracers	Stability	Half-life	Partition coefficient	Tumor uptake at 1 h p.i.	References
[^{68}Ga]Ga-FAPI-04	Above 95% after 4 h	Short	−3.52 (Log*P*)	3.00 ± 0.36 %ID/g at 20 min p.i. SKOV3 tumor mice	*Molecular pharmaceutics* 20, no. 5 (2023): 2402-2414 [1].

					Journal of Medicinal Chemistry 67, no. 19 (2024): 17785-17795 [2].
$[^{68}Ga]Ga$-(FAPI-04)$_2$	Above 99% after 3 h	Short	−3.05 (Log*P*)	6.80 ± 0.89 %ID/g, at 3 h p.i. SKOV3 tumor mice	*Molecular pharmaceutics* 20, no. 5 (2023): 2402-2414 [1].
$[^{68}Ga]Ga$-FAPI-46	Above 95.3 % after 3 h	Short	−3.57 (Log*P*)	1.28 ± 0.40 %ID/g, HT1080-hFAP tumor bearing mice	*European Journal of Nuclear Medicine and Molecular Imaging* (2022): 1-11 [3]. *Pharmaceuticals* 16, no. 8 (2023): 1138 [4].
$[^{68}Ga]Ga$-OncoFAP-DOTAGA	No degradation up to 2 h (no data beyond that)	Short	−3.91 ($LogD_{7.5}$)	2.49 ± 0.56 %ID/g, HT1080-hFAP tumor bearing mice	*European Journal of Nuclear Medicine and Molecular Imaging* (2022): 1-11 [3].
$[^{68}Ga]Ga$-FAPI-02	Above 99% after 24 h	Short	-	4.51 ± 0.816 %ID/g, HT-1080-FAP tumor xenografts	*Journal of Nuclear Medicine* 59, no. 9 (2018): 1423-1429 [5].
$[^{18}F]$-FAPI-74	No degradation up to 4 h (no data beyond that)	Mod erate	−2.42 ($LogD_{7.5}$)	6.9 %ID/g after 30 min, HT-1080-FAP bearing mice	*EJNMMI Radiopharmacy and Chemistry* 6, no. 1 (2021): 28 [6].
$[^{18}F]$AlF-FAP-NUR	Above 90% after 4 h	Mod erate	−2.45 (Log*P*)	6.67 ± 2.96 %ID/g, 293T-FAP tumor-bearing mice	*EJNMMI research* 14, no. 1 (2024): 87 [7].
$[^{64}Cu]Cu$-CB-TE2A-FAPI-04	Above 99% after 24 h	Long	−1.60 (Log*P*)	1.4 ± 0.5 %ID/g, XP-185 tumorgrafts	This work
$[^{64}Cu]Cu$-CB-TE2A-(FAPI-04)$_2$	Above 99% after 24 h	Long	−1.20 (Log*P*)	5.3 ±1.4 %ID/g, XP-185 tumorgrafts. 6.25 ± 1.87% ID/g at 4 h p.i., RENCA-FAP tumors.	This work

References:

1. Zhong, X.; Guo, J.; Han, X.; Wu, W.; Yang, R.; Zhang, J.; Shao, G. Synthesis and preclinical evaluation of a novel FAPI-04 dimer for cancer theranostics. *Molecular pharmaceutics* **2023**, *20*, 2402-2414.
2. Huang, J.; Zhang, X.; Liu, Q.; Gong, F.; Huang, Y.; Huang, S.; Fu, L.; Tang, G. 68Ga/177Lu-labeled theranostic pair for targeting fibroblast activation protein with improved tumor uptake and retention. *Journal of Medicinal Chemistry* **2024**, *67*, 17785-17795.
3. Backhaus, P.; Gierse, F.; Burg, M.C.; Büther, F.; Asmus, I.; Dorten, P.; Cufe, J.; Roll, W.; Neri, D.; Cazzamalli, S. Translational imaging of the fibroblast activation protein (FAP) using the new ligand [68 Ga] Ga-OncoFAP-DOTAGA. *European Journal of Nuclear Medicine and Molecular Imaging* **2022**, 1-11.
4. Plhak, E.; Pichler, C.; Dittmann-Schnabel, B.; Gößnitzer, E.; Aigner, R.M.; Stanzel, S.; Kvaternik, H. Automated synthesis of [68Ga] Ga-FAPI-46 on a Scintomics GRP synthesizer. *Pharmaceuticals* **2023**, *16*, 1138.
5. Loktev, A.; Lindner, T.; Mier, W.; Debus, J.; Altmann, A.; Jäger, D.; Giesel, F.; Kratochwil, C.; Barthe, P.; Roumestand, C. A tumor-imaging method targeting cancer-associated fibroblasts. *Journal of Nuclear Medicine* **2018**, *59*, 1423-1429.
6. Naka, S.; Watabe, T.; Lindner, T.; Cardinale, J.; Kurimoto, K.; Moore, M.; Tatsumi, M.; Mori, Y.; Shimosegawa, E.; Valla Jr, F. One-pot and one-step automated radio-synthesis of [18F] AlF-FAPI-74 using a multi purpose synthesizer: a proof-of-concept experiment. *EJNMMI Radiopharmacy and Chemistry* **2021**, *6*, 28.
7. Liu, S.; Zhang, Z.; Zhong, J.; Zhong, H.; Fu, Y.; Liu, L.; Ye, X.; Wang, X. Preclinical evaluation and first-in-human study of [18F] AlF-FAP-NUR for PET imaging cancer-associated fibroblasts. *EJNMMI research* **2024**, *14*, 87.
